# *Akkermansia muciniphila* in Metabolic Disease: Far from Perfect

**DOI:** 10.3390/ijms262311602

**Published:** 2025-11-29

**Authors:** Oana Laura Mierlan, Camelia Busila, Octavian Amaritei, Dogaru Elena, Cosmin Raducu Raileanu, Nicoleta-Maricica Maftei, Madalina Nicoleta Matei, Gabriela Gurau

**Affiliations:** 1 Faculty of Medicine and Pharmacy, “Dunarea de Jos” University of Galati, 800008 Galati, Romania; laura.mierlan@ugal.ro (O.L.M.); camelia.busila@ugal.ro (C.B.); cosmin.raileanu@ugal.ro (C.R.R.); madalina.matei@ugal.ro (M.N.M.); gabriela.gurau@ugal.ro (G.G.); 2 “Sf. Ioan” Emergency Clinical Pediatric Hospital, 800487 Galati, Romania; 3 “Sf. Andrei” Clinical Emergency County Hospital, 800578 Galati, Romania; 4 Center for Research and Technology Transfer in the Medico-Pharmaceutical Field, “Dunarea de Jos” University of Galati, 800008 Galati, Romania

**Keywords:** *A. muciniphila*, microbiome, metabolic disease, obesity, diabetes

## Abstract

The root of metabolic disease lies in the gastrointestinal tract, where nutrient absorption occurs. Within this environment, a diverse community of microorganisms exerts effects that extend beyond the intestinal barrier. *Akkermansia muciniphila* (*A. muciniphila*), one such bacterium, has been associated with enhanced intestinal integrity, reduced intestinal inflammation, weight loss, and improved insulin sensitivity, mediated through mucus fermentation, production of short-chain fatty acids (SCFAs), bacterial proteins, and extracellular vesicles (EVs). Research in this field is promising, yet far from perfect. Its clinical applicability remains limited by factors such as strain variability, scarcity of human intervention studies, and the lack of established causality. While associations have been consistently observed in both rodent and human studies, causality has thus far been demonstrated only in animal models. This issue is of critical importance, as metabolic disease remains highly prevalent, carries systemic consequences, and imposes a substantial burden on healthcare systems, underscoring the urgent need for alternative therapeutic strategies. The aim of this narrative review is to synthesize current knowledge on *A. muciniphila* and to highlight the key limitations consistently reported in the literature. By addressing these factors, the review seeks to provide realistic perspectives on its therapeutic potential and to outline directions for future research.

## 1. Introduction

Obesity and diabetes mellitus collectively constitute a substantial global health burden, contributing to the escalating prevalence of metabolic, cardiovascular, and neoplastic disorders [1,2,3]. The incidence of both conditions continues to rise worldwide, driven largely by sedentary behaviors and chronic caloric excess [4]. Moreover, current therapeutic approaches have varying effectiveness in halting their progression at the population level, highlighting the need for alternative or adjuvant therapies capable of mitigating their consequences [5,6].

The intestinal microbiome—comprising a diverse community of microorganisms including bacteria, viruses, and fungi—interacts with the gastrointestinal tract through a wide range of metabolites, forming a highly complex ecosystem [7,8]. This ecosystem depends on a delicate balance to maintain dynamic symbiosis between microbial populations and the human host [9]. Within this context, *A. muciniphila*, a Gram-negative bacterium naturally present in the human gut, has been repeatedly associated with improved metabolic profiles, including enhanced insulin sensitivity, weight regulation, and reduced inflammation [10].

Despite these promising associations, the clinical translation of *A. muciniphila* remains “far from perfect”—current evidence remains largely correlational, and numerous limitations constrain its applicability in clinical practice, such as strain variability, the scarcity of human studies, the lack of established causality in humans, and the methodological challenges that complicate interpretation [11]. In other words, although preclinical and associative human data suggest potential benefits, significant gaps in evidence and practical hurdles prevent its reliable application in clinical practice.

The aim of this narrative review is to critically assess the metabolic benefits attributed to *A. muciniphila* while explicitly highlighting the limitations that underpin its “far from perfect” status. By integrating these considerations, the review seeks to provide realistic perspectives on therapeutic potential and to guide future research directions.

## 2. A Symbiotic Relationship

A symbiotic relationship is established between the host and the intestinal microbiota, and this interaction is mediated primarily through the intestinal barrier—a complex, multilayered defense system located at the interface between the external environment and the internal milieu [12,13]. The first line of defense is the physical barrier formed by mucus, which is secreted by goblet cells that belong to a tightly connected epithelial layer bound together by tight junctions [14]. This mucus nourishes beneficial bacteria, which are part of the microbiome, while protecting against the translocation of pathogenic microorganisms [15]. Below the mucus layer lies the epithelial barrier, which functions as the second line of defense and cooperates with resident immune cells—including dendritic cells, macrophages, B cells, and T cells—to regulate host protection. Through coordinated interaction, these cells contribute to the recognition and neutralization of harmful pathogens and to maintain mucosal immune homeostasis [12]. In pathological conditions such as intestinal inflammation, autoimmune diseases, and cancer, the junctions between intestinal epithelial cells can become compromised, facilitating the passage of harmful bacteria and their toxins across the intestinal wall into the bloodstream, thereby promoting systemic inflammation [16,17,18,19].

### 2.1. A. muciniphila-Taxonomy and General Characteristics

Isolated first from human fecal matter in 2004, *A. muciniphila,* the most well-characterized member of the phylum *Verrucomicrobia*, has been confirmed by multiple taxonomic and genomic studies as an anaerobic, Gram-negative bacterium, a mucin-degrading bacterium with a low tolerance to oxygen, that constitutes approximately 3% (1% to 4%) of the gut microbial population [20,21]. Its colonization of the gut starts during the first year of life, through breast milk, and its presence has been documented in all life stages. It is mostly found on the outer mucus layer of the intestinal tract, and it’s most abundant in the caecum [22]. Genomic analyses have revealed substantial intra-species diversity, identifying multiple phylogroups or lineages within *A. muciniphila* [23].

### 2.2. Mucus Degradation

The intestinal mucus layer is composed primarily of water, inorganic ions, and mucin glycoproteins, among which MUC2 represents the predominant structural component. *A. muciniphila* is highly specialized to utilize mucin as a principal carbon and nitrogen source. This process is enabled by an extensive enzymatic repertoire, including glycoside hydrolases (GHs), sulfatases, and polysaccharide lyases (PLs), which collectively remove sulfate residues, cleave O-glycosidic bonds, and depolymerize the mucin matrix [22]. The metabolic degradation of mucin yields monosaccharides, oligosaccharides, and SCFAs—notably acetate and propionate—which function as key microbial-derived metabolites with effects on both host physiology and the surrounding microbiota. Enzymatic turnover of mucin also promotes compensatory mucus secretion by goblet cells, thereby contributing to sustained epithelial barrier renewal. Beyond its metabolic activity, *A. muciniphila* exerts direct regulatory effects on epithelial homeostasis. Through ADP-heptose–dependent activation of the alpha kinase 1/TRAF-interacting protein with FHA domain (ALPK1/TIFA) pathway, it upregulates MUC2, as well as the anti-apoptotic genes baculoviral IAP repeat-containing protein 3 (BIRC3) and tumor necrosis factor alpha–induced protein 3 (TNFAIP3), enhancing epithelial cell viability and limiting paracellular permeability [22,24]. These actions have been associated with attenuation of lipopolysaccharide (LPS)-induced low-grade inflammation and improved insulin sensitivity. Furthermore, *A. muciniphila* reinforces barrier integrity by increasing the expression of major tight-junction proteins, including ZO-1 (zonula ocludens-1), occludin, and claudin-3, thereby strengthening mucosal cohesion [22,24]. However, this activity must be tightly regulated, as both excessive degradation and mucus accumulation can lead to thinning or thickening of the mucus layer, which promotes inflammation, bacterial translocation, and colorectal pathology [25]. Experimental studies have shown that high densities of *A. muciniphila* can reduce mucus content and compromise barrier function, while moderate levels maintain a balanced mucus turnover, highlighting the importance of enzymatic regulation for intestinal homeostasis [26,27,28].

### 2.3. Metabolic Signaling via SCFAs

Dietary undigested carbohydrates, mucin glycoproteins, and fibers are fermented by gut bacteria, producing metabolites such as trimethylamine and SCFAs. Among these microbes, *A. muciniphila* is a major contributor to SCFA production, particularly acetate and propionate, through the fermentation of mucin and dietary polysaccharides [11,29,30]. These SCFAs serve as energy substrates for colonocytes, support epithelial integrity, and modulate immune responses by interacting with intestinal epithelial and immune cells. Such effects are mediated, in part, through G-protein-coupled receptors (GPR41 and GPR43), which are expressed on enterocytes, colonocytes, enteroendocrine cells, neutrophils, and neurons [31]. Accumulating evidence indicates that *A. muciniphila* abundance correlates with SCFA levels and beneficial metabolic outcomes [32,33,34]. SCFAs support metabolic health by slowing gastric emptying, increasing satiety, and promoting insulin secretion through the stimulation of intestinal L cells to produce incretin hormones such as glucagon-like peptide-1 (GLP-1), the same peptide targeted by antidiabetic drugs that have proven effective for weight loss Although the magnitude of SCFA-induced GLP-1 secretion relative to pharmacological interventions has not been directly compared, accumulating evidence from over 50 experimental and clinical studies highlights their significant role in energy homeostasis, glucose regulation, and metabolic health [32,35,36].

### 2.4. Amuc Proteins: Molecular Mediators

**Figure 1:**
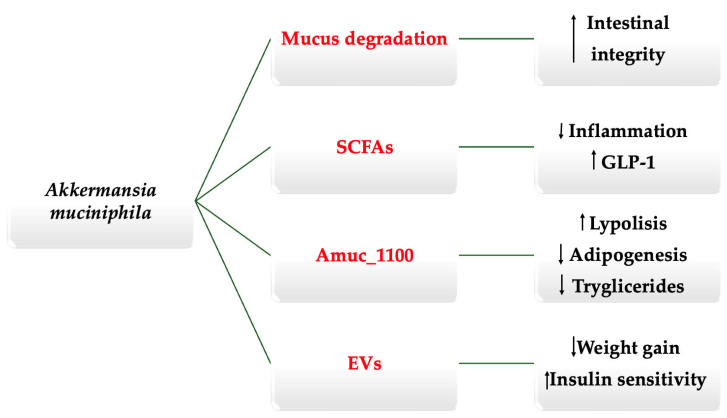
Schematic representation of the key mechanisms associated with *A. muciniphila* (summarized from the concepts discussed in the text). Up arrow = Increased; Down arrow = decreased.

Bacteria are known to produce various toxins and proteins that influence the host in diverse ways, and *A. muciniphila* is no exception [37,38]. This species secretes a family of proteins that have recently been studied in isolation, including Amuc_1100, Amuc_1434, and Amuc_1409, which act as signaling molecules mediating several of the bacterium’s beneficial effects [11,39,40,41]. Animal studies have demonstrated that these proteins exert favorable metabolic and intestinal outcomes even when administered independently, following bacterial pasteurization. Zheng et al. [42] reported that Amuc_1100, a protein derived from *A. muciniphila*, promotes the browning of adipocytes by upregulating genes such as peroxisome proliferator-activated receptor γ (PPAR-γ), peroxisome proliferator-activated receptor gamma coactivator 1α (PGC1α), and fibroblast growth factor 21 (FGF21), while enhancing fatty acid β-oxidation via acyl-CoA oxidase 1 (Acox1). These molecular changes result in increased lipolysis and suppressed adipogenesis. Collectively, these findings suggest that Amuc_1100 mediates a substantial portion of the anti-obesity effects observed with *A. muciniphila* supplementation, underscoring its central role in the bacterium’s metabolic benefits. Similarly, Kang et al. [39] showed that Amuc_1409 protects against intestinal injury by promoting intestinal stem cell proliferation and epithelial renewal, while Meng et al. [40] found that Amuc_1434 inhibits the growth of colorectal neoplastic cells. These findings are promising, as they suggest the possibility of isolating specific proteins to achieve targeted therapeutic effects. Such an approach could theoretically retain the beneficial outcomes observed with *A. muciniphila*, presented in Figure 1, while minimizing or avoiding the potential adverse effects hypothesized with an excess of whole-bacterium administration, such as inflammatory bowel disease and neurodegenerative disorders [43,44,45,46]. In addition, *A. muciniphila* secretes P9-protein, which modulates host metabolism by binding to intercellular adhesion molecule-2 (ICAM-2), enhancing uncoupling protein-1 (UCP-1)–dependent thermogenesis in brown adipose tissue and stimulating GLP-1 secretion from intestinal L cells. Through these pathways, P9 may indirectly support redox homeostasis and reduce oxidative stress, although direct evidence of its effects on oxidative stress regulatory mechanisms is still limited [https://link.springer.com/article/10.1186/s12967-025-07149-z] (accessed on 25 November 2025).

### 2.5. A. muciniphila Abundance: Diet, Aging, and Antibiotics

Fluctuations in the abundance of *A. muciniphila* have been observed in association with aging, dietary changes, intestinal and systemic diseases, and antibiotic use [11]. Currently, its assessment relies on molecular techniques such as 16S rRNA gene sequencing, shotgun metagenomics, and metatranscriptomics; however, these methods have notable limitations, as high stool concentrations do not necessarily correlate with effective mucosal colonization [11,47,48,49].

Caloric restriction, along with supplements such as pomegranate extract, resveratrol, polydextrose, sodium butyrate, and inulin, has been reported to raise *A. muciniphila* levels, while diets low in fermentable carbohydrates tend to reduce them [50,51,52]. Overall, greater intake of dietary fibers—particularly soluble fibers like inulin—consistently supports the abundance of *A. muciniphila* [53].

The relationship between antibiotics and *A. muciniphila* is nuanced, and current literature depicts a complex and sometimes contradictory landscape. Broad-spectrum antibiotics do not distinguish between harmful and beneficial bacteria, and their use is commonly associated with dysbiosis [54,55]. Nonetheless, *A. muciniphila* has demonstrated resistance to fluoroquinolones [56]. Han et al. [57] reported in a 2025 study that penicillin exposure may select for specific *A. muciniphila* variants in the gut, while simultaneously diminishing the bacterium’s metabolic benefits in diet-induced obesity models. In contrast, Liu et al. [58] found that administration of Amuc_1100 reduced intestinal inflammation and improved symptoms in a mouse model of acute antibiotic-associated diarrhea induced by lincomycin and ampicillin, compared with controls.

Becken et al. [59] cultured mucophilic bacteria from 123 fecal samples collected from 49 donors and isolated 71 new strains of *A. muciniphila*. These strains differed in their growth patterns, oxygen utilization, affinity for epithelial cells, and secretion of various metabolites [60,61]. Such variability may explain why some studies report inconsistent metabolic effects, with a few even suggesting potential adverse associations, such as an increased risk of colon cancer or neurodegenerative diseases [62,63]. These findings highlight the need for further research to identify which strains exert beneficial effects and which are most suitable for specific pathological contexts.

## 3. *A. muciniphila* in Obesity: Insights and Evidence

### 3.1. Animal Studies on Obesity and Human Studies on Obesity

Obesity lies at the base of a pyramid, supporting other conditions such as diabetes, atherosclerosis, and neoplastic diseases that build upon it, ultimately leading to the apex, where morbidity and mortality reside [64,65,66]. Dietary interventions, pharmacological treatments, and surgical approaches currently represent the main therapeutic strategies, each with varying degrees of effectiveness [6]. However, treatment failure is often driven by psychological factors, including the influence of highly processed, addictive foods on appetite and food preferences, which ultimately affect energy balance [67].

The influence of the microbiome has recently been explored, yielding promising results and suggesting that caloric intake may not be the sole dietary factor capable of modulating body weight [68]. The complexity and relative novelty of this field necessitated extensive research to elucidate the intricate interactions between bacteria and the host. The inherently invasive nature of early experimental approaches positioned animal studies at the forefront of this research, enabling investigators to determine whether sufficient mechanistic evidence existed to justify further exploration in humans.

Liu et al. [69] administered the antioxidant quercetin to a mouse model of obesity to evaluate its effects on metabolic parameters. Mice fed a high-fat diet in the intervention group showed a significant increase in the *A. muciniphila* population, which was associated with a reduction in body weight and fat mass percentage compared to mice on the same diet that did not receive the supplement. In the second part of the study, the authors examined whether these effects could be replicated by supplementing the mice for 10 weeks with *A. muciniphila* itself. Similar results were observed, with reductions in body weight and improvements in metabolic parameters such as insulin sensitivity. These results were also reproduced by Yang et al. [70], in which a 12-week supplementation with *A. muciniphila* led to reduced low-grade inflammation, preservation of the intestinal barrier—as demonstrated by histological analysis—and prevention of hepatic lipid accumulation, along with decreases in body weight and improved insulin sensitivity.

Everard et al. [71] showed that mice with obesity and type 2 diabetes had a lower abundance of *A. muciniphila*, which increased following prebiotic administration. Moreover, direct supplementation with *A. muciniphila* once again resulted in an improved metabolic profile. In contrast, the group receiving heat-killed *A. muciniphila* did not exhibit the same benefits, leading the authors to conclude that bacterial viability is essential for its effects. However, results remain inconclusive regarding this aspect, as other studies have reported comparable benefits following the administration of pasteurized bacteria [72,73], and Plovier et al. [74] even demonstrated an enhanced efficiency for the administration of pasteurized *A. muciniphila* in mice, with effects partially mediated by the bacterial membrane protein Amuc_1100, which interacts with host Toll-like receptor 2 (TLR2) receptors, modulating metabolism without requiring live bacteria.

### 3.2. Human Studies on Obesity

Fatty acid β-oxidation is impaired in individuals with obesity as a consequence of the reduced capacity of both the liver and skeletal muscle to oxidize stored energy [75]. This impairment increases the demand for additional glucose intake, which is subsequently stored rather than properly oxidized for energy. Acylcarnitines are compounds that rise in the bloodstream as a result of fatty acid oxidation [76]. In subjects undergoing plasma sampling before and after exercise, acylcarnitine levels increase markedly, reflecting enhanced fatty acid oxidation [77]. In a study by Depommier et al. [78], acylcarnitine concentrations were found to increase in the intervention group that received a supplement containing *A. muciniphila*, suggesting that supplementation mimics the metabolic effects of exercise. Notably, supplementation also promoted the utilization of ketone bodies as an energy source, as indicated by elevated levels of acetoacetate and 3-hydroxybutyrate. These ketone bodies are associated with a more favorable redox state, leading to reduced oxidative stress, inflammation, and enhanced carnitine/acylcarnitine-dependent fatty acid transport into mitochondria [79].

Cao et al. [80] randomized 40 overweight and obese subjects to receive either a Chinese herbal supplement or a placebo. Although the supplement did not contain probiotics, an increase in the abundance of *A. muciniphila* and *Enterococcus faecium*, along with a decrease in *Proteobacteria*, was observed. The primary outcome of the study was a reduction in body weight and BMI, which was significantly more pronounced in the intervention group—72.22% of participants in this group lost more than 5% of their body weight within three months, compared with 36.84% in the placebo group. The supplement also significantly reduced metabolic biomarkers, including low-density lipoprotein (LDL) cholesterol and blood glucose, to a greater extent than the placebo. Whether these beneficial changes resulted directly from the supplement itself or from the associated shifts in microbiome composition remains uncertain. A limitation of this study lies in the presence of multiple confounding factors: all participants received guidelines on exercise and healthy eating, and were encouraged to adopt lifestyle changes. Although these recommendations were provided to both groups, the lack of strict control over these variables limits the strength of the conclusions, as lifestyle remains the predominant determinant of weight management [65]. Nonetheless, this study contributes to a broader understanding in which *A. muciniphila* once again emerges as a marker of improved metabolic health [81,82].

## 4. *A. muciniphila* and Diabetes: Current Understanding

### 4.1. Animal Studies

Beyond its growing prevalence, diabetes carries a substantial health and economic burden, being a major risk factor for cardiovascular disease, renal failure, neuropathy, retinopathy, and other disabling complications that markedly impair quality of life [83]. Lifestyle factors—particularly diet—play a central role in both the development and management of diabetes, influencing glucose metabolism, insulin sensitivity, and systemic inflammation [84]. In recent years, attention has increasingly shifted toward the gut microbiome as a potential modulator of these processes [85]. Alterations in intestinal microbial composition have been associated with impaired glucose homeostasis and chronic inflammation, suggesting that targeting the microbiome through dietary interventions or supplementation may open new perspectives for the prevention and treatment of diabetes [86].

When LPS pass through the gaps between epithelial cells, they activate the nuclear factor-kB (NF-κB) and Jun N-terminal kinase (JNK) signaling pathways, which in turn promote both local and systemic inflammation [87,88]. By maintaining intestinal integrity, *A. muciniphila* helps counteract this translocation [89]. Furthermore, it regulates lipid and glucose metabolism by suppressing lipogenic genes, including sterol regulatory element-binding protein 1c (SREBP1c) and fatty acid translocase (CD36), thereby enhancing insulin sensitivity and overall metabolic efficiency [90]. A meta-analysis of 15 studies by Liu et al. [91] concluded that, in animal studies, *A. muciniphila* administration prevents the onset of type 2 diabetes and obesity.

EVs are lipid bilayer structures secreted by gut microbiota. Many bacterial species continuously release these spherical vesicles, which contain a diverse array of components, including proteins, lipids, nucleic acids, and lipopolysaccharides [92]. Far from being inert, EVs exert biological effects that can be either beneficial or harmful, depending on their bacterial origin [92,93]. Choi et al. [94] demonstrated that in vivo administration of stool-derived EVs from high-fat diet (HFD)-fed mice induced insulin resistance and glucose intolerance compared with those from regular diet (RD)-fed mice. Notably, *Pseudomonas panacis*-derived EVs disrupted insulin signaling in both skeletal muscle and adipose tissue. Conversely, Chelakkot et al. [95] reported that fecal samples from healthy individuals contained higher levels of *A. muciniphila*-derived EVs (AmEVs) than those from patients with type 2 diabetes, and in HFD-induced diabetic mice, the administration of AmEVs improved tight junction integrity, reduced weight gain, and enhanced glucose tolerance. Furthermore, while AmEVs decreased gut permeability, *Escherichia coli*-derived EVs showed no such effect, highlighting the species-specific functionality of bacterial EVs.

### 4.2. Human Studies

Depommier et al. [96] published a randomized double-blind placebo-controlled pilot study in 2019 that investigated the effect of a 3-month supplementation with *A. muciniphila* in overweight and obese individuals with insulin resistance but without diabetes. Even though the number of participants was low, *A. muciniphila* significantly improved insulin sensitivity compared to placebo. Compared to baseline, plasma LPS and the activity of dipeptidyl peptidase-IV (DPP-IV) decreased in the intervention group, suggesting two potential mechanisms for the decreased insulin resistance: decreased endotoxemia and increased glucagon-like peptide 1 (GLP-1), respectively. Furthermore, the supplementation was safe and did not change the overall microbiome at the end of the study compared to baseline. Further studies with a higher number of subjects are needed to assess whether the outcomes will be the same.

After a 12-week administration of a probiotic containing *A. muciniphila*, Perraudeau et al. [97] observed in a small cohort of diabetic subjects that, although there were no changes in body weight, lipid parameters, homeostatic model assessment (HOMA) index, or inflammatory markers such as C-reactive protein (CRP), interleukin-6 (IL-6), and tumor necrosis factor-alpha (TNF-α), postprandial glucose handling improved, with a significant decrease in glucose area under the curve (AUC). The study has several limitations, the most notable being the inclusion of heterogeneously treated subjects, some of whom were receiving metformin or sulfonylureas. This variability complicates the interpretation of the findings and may limit their applicability to the general population.

**Figure 2:**
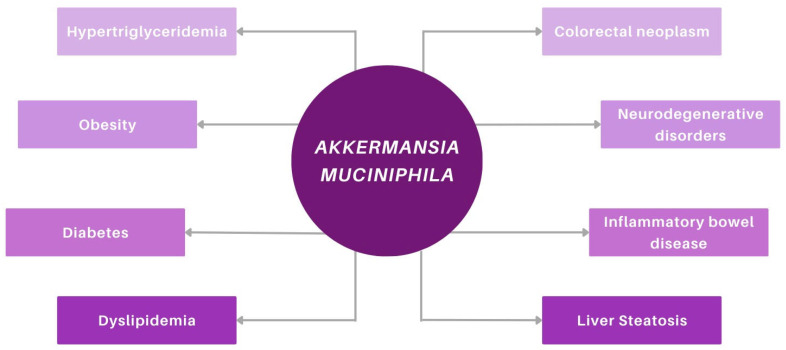
Implications of *Akkermansia muciniphila* in various diseases (discussed in the text).

Wang et al. [98] showed that in subjects with type 1 diabetes, the addition of a probiotic supplement to conventional insulin therapy enriched the intestinal microbial composition, with increases in *Bifidobacterium animalis*, *Lactobacillus salivarius*, and *A. muciniphila* compared to insulin therapy alone. These changes were accompanied by a decrease in pro-inflammatory cytokines such as IL-8, IL-17, and TNF-α, as well as an improvement in glycemic control that persisted even three months after discontinuing the intervention. Although type 1 and type 2 diabetes have distinct pathophysiological mechanisms, the observation that alterations in microbial composition appear to influence both suggests a more intricate interplay between inflammation, the immune system, and metabolic regulation. At the core of this interaction lies the gut microbiome, which exerts regulatory effects across all three systems. Figure 2 illustrates the associations between *A. muciniphila* and various human diseases, including metabolic, neurodegenerative, and gastrointestinal disorders.

## 5. Current Limitations

Despite the promising findings regarding *A. muciniphila*, several limitations should be considered when interpreting the results (See Box 1). These constraints highlight areas where further research is needed to validate the observed effects and to translate preclinical and early clinical evidence into robust, generalizable conclusions. First, many clinical studies involve small sample sizes, limiting statistical power and generalizability. Second, heterogeneity in participant characteristics, including concurrent medications, dietary habits, and metabolic status, complicates the interpretation of results. Third, most studies rely on stool abundance as a proxy for intestinal colonization, which may not accurately reflect the bacterium’s activity or distribution along the gastrointestinal tract. Fourth, the optimal dosing, formulation, and duration of supplementation remain undefined, and long-term safety data are limited. Finally, mechanistic pathways in humans are still not fully elucidated, emphasizing the need for comprehensive studies integrating clinical, microbial, and molecular analyses.

Although evidence supports the beneficial effects of *A. muciniphila*, its overgrowth may, in certain contexts, enhance susceptibility to enteric infections by promoting pathogen virulence, as shown in mouse models where elevated levels were associated with increased intestinal galactosylation and reduced group 3 innate lymphoid cells (ILC3s), resulting in higher *Citrobacter rodentium* virulence [https://www.nature.com/articles/s41564-025-01933-9] (accessed on 25 November 2025).

Box 1 **Limitations regarding *A. muciniphila.***

- Absence of human studies demonstrating causality
- Limited translational value of rodent findings
- Uncontrolled lifestyle-related confounders
- Insufficient evidence on the efficacy of *A. muciniphila* supplementation
- Indirect assessment, as stool abundance may not reflect colonic levels
- Strain variability, with heterogeneous effects among *A. muciniphila* isolates
- Potential safety concerns
- Influence of host genetics on microbial colonization

## 6. Materials and Methods

### 6.1. Research Strategy

Keywords such as “*A. muciniphila*,” “Obesity,” “Diabetes,” “Metabolic disease,” “Microbiome,” “Short chain fatty acids,” along with various Boolean combinations of these terms, were used to conduct a comprehensive literature search across PubMed, ScienceDirect, Google Scholar, and journals such as MDPI and Frontiers. Animal studies were reviewed first, followed by human studies, with all publications from 2015 to 2025 included to ensure the novelty of the evidence. The Section 1 of this review focuses on the microbiome and the unique characteristics of *A. muciniphila*, while the second synthesizes data from both animal and human studies. Given the narrative nature of this review, no formal risk of bias tools were applied. References were organized using Zotero, and artificial intelligence tools were employed to check grammar and ensure clarity and coherence of the text.

### 6.2. Inclusion and Exclusion Criteria

Study selection followed predefined inclusion and exclusion criteria. Only peer-reviewed studies examining the microbiome, *A. muciniphila*, and their relevance to metabolic disease were included. Exclusion criteria comprised non-peer-reviewed materials, duplicate publications, studies with insufficient or incomplete data, and studies published outside the predetermined time interval.

### 6.3. Study Selection

The initial search yielded 2020 results. After applying filters for publication timeframe and full-text availability, 1375 records remained. Following title and abstract screening, 96 studies were selected for inclusion based on relevance and novelty. Additionally, two further studies published outside the specified timeframe (2004 and 2013) were included, due to their foundational contributions to understanding lipolysis and the differential effects of live versus pasteurized *A. muciniphila*. These studies were deemed essential for providing historical and mechanistic context.

## 7. Conclusions

The association between *A. muciniphila* and improvements in metabolic health has been replicated in numerous studies; however, while correlations have been observed in both rodent and human research, causation has only been partially demonstrated in the former. Establishing causality in humans may be virtually impossible, as it would require strict control of all dietary and lifestyle factors. Even if a causal role for *A. muciniphila* is confirmed, supplementation would still need to be rigorously tested for efficacy, particularly considering the potential side effects discussed in previous sections. Limitations in assessment methods may also affect results, as the stool abundance of *A. muciniphila* does not necessarily reflect its presence throughout the gut, given that some bacteria may transit without colonizing. Future studies should prioritize large, controlled trials, optimized dosing strategies, and mechanistic investigations to clarify its effects on glucose metabolism, insulin sensitivity, and gut barrier function. Standardized monitoring of gut colonization and activity will be essential to translate microbiome-based therapies into clinical practice.

## Data Availability

The original contributions presented in this study are included in the article. Further inquiries can be directed to the corresponding authors.

## Abbreviations

The following abbreviations are used in this manuscript:

SCFAs	Short-chain fatty acid
GLP-1	Glucagon-like peptide-1
rRNA	Ribosomal ribonucleic acid
LDL	Low-density lipoprotein
LPS	Lipopolysaccharides
NF-κB	Nuclear factor-kB
JNK	Jun N-terminal kinase
SREBP1c	Element-binding protein 1c
CD36	Fatty acid translocase
EVs	Extracellular vesicles
HFD	High-fat diet
AmEVs	*A. muciniphila*-derived EVs
RD	Regular diet
DPP-IV	Dipeptidyl peptidase-IV
HOMA	Homeostatic model assessment
CRP	C-reactive protein
IL-6	Interleukin-6
TNF-α	Tumor necrosis factor-alpha
AUC	Area under the curve
IL-8	Interleukin-8
IL-17	Interleukin-17
